# Ferrous Ion Alleviates Lipid Deposition and Inflammatory Responses Caused by a High Cottonseed Meal Diet by Modulating Hepatic Iron Transport Homeostasis and Controlling Ferroptosis in Juvenile *Ctenopharyngodon idellus*

**DOI:** 10.3390/antiox12111968

**Published:** 2023-11-06

**Authors:** Hengchen Liu, Shiyou Chen, Yan Lin, Wenqiang Jiang, Yongfeng Zhao, Siyue Lu, Linghong Miao, Xianping Ge

**Affiliations:** 1 Wuxi Fisheries College, Nanjing Agricultural University, Wuxi 214081, China; lhcffrc@sina.cn (H.L.); chen-sy@cau.edu.cn (S.C.); 2020213006@stu.njau.edu.cn (W.J.); zhaoyf@ffrc.cn (Y.Z.); gexp@ffrc.cn (X.G.); 2 Key Laboratory of Freshwater Fisheries and Germplasm Resources Utilization, Ministry of Agriculture and Rural Affairs, Freshwater Fisheries Research Center, Chinese Academy of Fishery Sciences, Wuxi 214081, China; liny@ffrc.cn (Y.L.); lusiyue@ffrc.cn (S.L.)

**Keywords:** herbivorous fish, plant protein source, ion transporter, lipid metabolism, inflammation

## Abstract

To investigate the mechanisms through which ferrous ion (Fe^2+^) addition improves the utilization of a cottonseed meal (CSM) diet, two experimental diets with equal nitrogen and energy content (low-cottonseed meal (LCM) and high-cottonseed meal (HCM) diets, respectively) containing 16.31% and 38.46% CSM were prepared. Additionally, the HCM diet was supplemented with graded levels of FeSO_4_·7H_2_O to establish two different Fe^2+^ supplementation groups (HCM + 0.2%Fe^2+^ and HCM + 0.4%Fe^2+^). Juvenile *Ctenopharyngodon idellus* (grass carps) (5.0 ± 0.5 g) were fed one of these four diets (HCM, LCM, HCM + 0.2%Fe^2+^ and HCM + 0.4%Fe^2+^ diets) for eight weeks. Our findings revealed that the HCM diet significantly increased lipid peroxide (LPO) concentration and the expression of lipogenic genes, e.g., sterol regulatory element binding transcription factor 1 (*srebp1*) and stearoyl-CoA desaturase (*scd*), leading to excessive lipid droplet deposition in the liver (*p* < 0.05). However, these effects were significantly reduced in the HCM + 0.2%Fe^2+^ and HCM + 0.4%Fe^2+^ groups (*p* < 0.05). Plasma high-density lipoprotein (HDL) concentration was also significantly lower in the HCM and HCM + 0.2%Fe^2+^ groups compared to the LCM group (*p* < 0.05), whereas low-density lipoprotein (LDL) concentration was significantly higher in the HCM + 0.2%Fe^2+^ and HCM + 0.4%Fe^2+^ groups than in the LCM group (*p* < 0.05). Furthermore, the plasma levels of liver functional indices, including alkaline phosphatase (ALP), aspartate aminotransferase (AST), alanine aminotransferase (ALT), and glucose (GLU), were significantly lower in the HCM + 0.4%Fe^2+^ group (*p* < 0.05). Regarding the expression of genes related to iron transport regulation, transferrin 2 (*tfr2*) expression in the HCM group and Fe^2+^ supplementation groups were significantly suppressed compared to the LCM group (*p* < 0.05). The addition of 0.4% Fe^2+^ in the HCM diet activated *hepcidin* expression and suppressed ferroportin-1 (*fpn1*) expression (*p* < 0.05). Compared to the LCM group, the expression of genes associated with ferroptosis and inflammation, including acyl-CoA synthetase long-chain family member 4b (*acsl4b*), lysophosphatidylcholine acyltransferase 3 (*lpcat3*), cyclooxygenase (*cox*), interleukin 1β (*il-1β*), and nuclear factor kappa b (*nfκb*), were significantly increased in the HCM group (*p* < 0.05), whereas Fe^2+^ supplementation in the HCM diet significantly inhibited their expression (*p* < 0.05) and significantly suppressed lipoxygenase (*lox*) expression (*p* < 0.05). Compared with the HCM group without Fe^2+^ supplementation, Fe^2+^ supplementation in the HCM diet significantly upregulated the expression of genes associated with ferroptosis, such as heat shock protein beta-associated protein1 (*hspbap1*), glutamate cysteine ligase (*gcl*), and glutathione peroxidase 4a (*gpx4a*) (*p* < 0.05), and significantly decreased the expression of the inflammation-related genes interleukin 15/10 (*il-15*/*il-10*) (*p* < 0.05). In conclusion, FeSO_4_·7H_2_O supplementation in the HCM diet maintained iron transport and homeostasis in the liver of juvenile grass carps, thus reducing the occurrence of ferroptosis and alleviating hepatic lipid deposition and inflammatory responses caused by high dietary CSM contents.

## 1. Introduction

The shortage of protein resources in animal feeding poses a significant challenge to the global livestock and aquaculture industries. In aquatic feed, fishmeal stands out as a high-quality protein source for aquatic animals due to its exceptional biological value, balanced amino acid composition, and digestibility [1]. However, with the rapid intensification of aquaculture, the disparity between the supply and demand for fishmeal in the aquafeed sector is on the rise. Therefore, substituting fishmeal with high-quality, cost-effective, and readily available plant protein sources has become an inevitable path to sustain the development of the aquaculture industry [1,2,3]. One strategy to promote the sustainable growth of aquaculture involves utilizing plant-derived byproducts to replace fishmeal. Cottonseed meal (CSM), a byproduct of cottonseed oil extraction, is widely used in aquatic feed due to its abundant availability, high protein content, and relatively low cost [4,5,6,7,8]. However, it contains anti-nutritional components that can be detrimental to animal health and performance if not appropriately managed, including free gossypol (FG), phytic acid, and tannins [9]. FG, a natural polyphenolic compound found in cottonseeds and other parts of the cotton plant, is the primary anti-nutritional factor that primarily accumulates in the liver [10]. FG negatively impacts growth, development, and overall physical health in fish, including hepatopancreatic injury, lipid metabolism disorders, and decreased autoimmune capacity [6,11].

Mitigating the growth inhibition and oxidative stress induced by CSM diets can be achieved through chemical detoxification, microbial fermentation, or supplementation with green additives, as previously demonstrated in juvenile grass carps (*Ctenopharyngodon idellus*) [12], Nile tilapia (*Oreochromis niloticus*) [13], and channel catfish (*Ictalurus punctatus*) [14]. Among these methods, chemical detoxification by adding FeSO_4_ to the CSM detoxification process has been widely adopted due to its cost-effectiveness, simplicity, and efficacy. The underlying principle involves the reaction of ferrous ions (Fe^2+^) with the aldehyde group of FG, forming a denatured gossypol-iron complex that renders FG inactive [15]. Previous experiments have shown that adding FeSO_4_ at a 1:1 ratio with FG alleviates the adverse effects of FG, leading to improved growth performance, feed utilization, and blood parameters [16]. Our previous study also demonstrated that a 1–2:1 ratio of Fe^2+^ to FG in a CSM diet promotes growth performance and enhances antioxidant capacity by improving intestinal health in the grass carp [7]. Subsequently, we observed a reduction in lipid peroxidation in the liver in response to Fe^2+^ supplementation in the high CSM (HCM) diet, which warrants further investigation.

Iron is an essential trace element crucial for supporting animal metabolism. However, excess free Fe^2+^ can harm cellular components by generating free radicals through redox reactions with various oxides [17]. Therefore, strict regulatory mechanisms are required to maintain intracellular iron content. Iron homeostasis is maintained via the regulation of intracellular and extracellular iron metabolism, including processes such as iron uptake, storage, and efflux. Dysfunction in iron homeostasis can result in ferroptosis due to oxidative stress [18,19,20]. Ferroptosis is a recently discovered mode of cell death distinct from apoptosis, necrosis, and autophagy, which is governed by various cellular processes, including iron levels and lipid metabolism [21]. The dynamic balance of intracellular iron content is a major driver of lipid peroxidation of polyunsaturated fatty acids (PUFAs) in phospholipids [22,23]. When cellular iron approaches saturation, excess Fe^2+^ accumulates within the cell, forming an unstable iron pool. Fe^2+^ then generates reactive oxygen species (ROS) via the Fenton reaction, triggering lipid oxidation and ultimately leading to ferroptosis [24]. Therefore, the disruption of intracellular iron metabolism and lipid peroxidation are critical events during ferroptosis. Furthermore, the cystine-glutamate reverse transporter (System Xc-) plays a crucial role in regulating ferroptosis [23]. System Xc- inhibition decreases intracellular cystine levels and triggers a glutathione (GSH) depletion, which indirectly inhibits the activity of glutathione peroxidase 4 (GPX4). This, in turn, leads to the accumulation of lipid peroxides and ultimately activates ferroptosis [25,26]. Additionally, ferroptosis is more immunogenic than apoptosis, as the former triggers inflammation by releasing damage-associated molecular patterns (DAMPs) from apoptotic cells [27].

The ability to utilize plant protein sources in herbivorous fish is usually stronger than in omnivorous and carnivorous fishes. Grass carps, as typical herbivorous fish, have an intestinal length of 2–5 times their body length. There is abundant secretion and high activity of amylase in the intestinal length, which is conducive to overcoming the difficulty of unfavorable digestion of plant-based protein sources. Therefore, feeding plant-based diets to grass carp, including CSM, is especially suitable. However, the growth performance and feed utilization efficiency in juveniles were significantly decreased when the CSM level in the diet exceeded 35.18% [28]. We previously showed that Fe^2+^ supplementation improves feed utilization of HCM (38% CSM) by alleviating the inflammatory response and regulating iron homeostasis to repair intestinal function [7]. Therefore, it was hypothesized that the additional supplementation of Fe^2+^ in the HCM diet modulated iron transport and ferroptosis, leading to attenuated hepatic lipid deposition and inflammatory responses.

Based on the above insights, we conducted further research to investigate the effects of Fe^2+^ supplementation in HCM-based diets on lipid metabolism and apoptosis in the grass carp, focusing on the regulatory mechanisms of ferroptosis.

## 2. Materials and Methods

### 2.1. Diet Preparation

Four isonitrogenous and isoenergetic diets (crude protein 38%, crude lipid 7%) were designed with different proportions of soybean meal, rapeseed meal, and CSM as the main protein sources. Four experimental feeds were prepared with the nutrient compositions summarized in Table 1. All diets were prepared as previously described [7]. In the low-CSM diet (LCM, containing 16.31% CSM), soybean meal and rapeseed meal were the main protein sources, whereas CSM replaced soybean meal in the HCM diet (containing 38.46% CSM). Additionally, the HCM diet was supplemented with 0.2% and 0.4% FeSO_4_·7H_2_O (denoted as HCM + 0.2%Fe^2+^ and HCM + 0.4%Fe^2+^, respectively).

### 2.2. Experimental Fish

The juvenile grass carp used in the experiment were obtained from the Nanquan Farming Base in the Freshwater Fisheries Research Center (FFRC) of the Chinese Academy of Fishery Sciences (CAFS). These fish were acclimatized in outdoor pond cages (4 m × 4 m × 1 m) for 15 days before the experiment to adapt to the experimental farming environment. A total of 360 similarly sized (initial weight 5.0 ± 0.5 g) and healthy juvenile grass carps were randomly assigned to the four experimental conditions in triplicate floating cages (1 m × 1 m × 1 m, 12 cages in total) and were fed one of the experimental diets for eight weeks. During the feeding trial, the juveniles were fed three times daily to apparent satiety, and the environmental conditions were maintained as follows: water temperature, 25–32.4 °C; pH, 7.0 ± 0.5; dissolved oxygen, 6 mg/L; light cycle 12 h and dark cycle 12 h. The fish were maintained under natural light conditions throughout the acclimation and experimental periods.

### 2.3. Sample Collection

At the end of the 8-week feeding trial, after a 36 h fast, the fish were anesthetized with MS-222, and blood samples were collected from the tail vein of three randomly selected fish using a sterile needle, after which the samples were placed in anticoagulation tubes. Following centrifugation at 4000 rpm for 10 min at 4 °C, the supernatant was collected and stored at −20 °C to quantify plasma biochemical indicators. Subsequently, liver tissues were promptly collected, flash-frozen in a liquid nitrogen tank, and then transferred for storage at −80 °C for lipid peroxide (LPO) and gene mRNA expression assays. Additionally, portions of the liver were fixed in 4% paraformaldehyde for Oil Red O staining.

### 2.4. Analysis of Plasma and Hepatic Indicators

Plasma low-density lipoprotein (LDL), high-density lipoprotein (HDL), total cholesterol (TC), glucose (GLU), total protein (TP), albumin (ALB), alkaline phosphatase (ALP), aspartate aminotransferase (AST), and alanine aminotransferase (ALT) were measured using a Shenzhen Myriad BS-400 automated biochemistry analyzer (Shenzhen Myriad Bio-Medical Electronic Co., Ltd., Shenzhen, China) with commercial reagent kits (Shanghai Zhicheng Biological Science and Technology Co., Ltd., Shanghai, China). Hepatic LPO was measured using commercial kits purchased from Nanjing Jiancheng Bioengineering Institute (Nanjing, China).

### 2.5. Oil Red O Staining of Liver Tissue

After fixation in 4% paraformaldehyde, frozen liver sections were prepared and incubated in Oil Red O staining solution for 10 min. Next, the sections were washed in 60% isopropanol and pure water. Hematoxylin re-staining was performed for 5 min, followed by differentiation, washing, counterstaining, and re-washing. The resulting slides were sealed with glycerol gelatin, and the morphology of liver lipid images was observed and captured using the Nikon Eclipse 80i microscope (Nikon, Tokyo, Japan). The lipid droplet area was calculated using the Image-Pro Plus 7.0 (Media Cybernetics Inc., Rockville, MD, USA). 

### 2.6. Determination of Relative Genes mRNA Expressions in the Liver

Total RNA was extracted using Trizol (TaKaRa Biomedical Technology Co., Ltd., Dalian, China), and RNA concentration and purity were assessed using a Nanodrop 2000 spectrophotometer (Thermo Science, Waltham, MA, USA). Reverse transcription to cDNA was performed using the PrimeScript RT reagent Kit with a gDNA Eraser (Takara Biomedical Technology Co., Ltd., Dalian, China). Quantitative reverse-transcription polymerase chain reaction (qRT-PCR) was conducted to determine relative genes mRNA expressions in the liver using Bio-Rad CFX96 (Hercules, CA, USA) with TB Green™ Premix Ex Taq™ II (TaKaRa Biomedical Technology Co., Ltd., Dalian, China). Table 2 summarizes the primer sequences for the target genes [*hepcidin*, ferroportin-1 (*fpn1*), *ferritin*, transferrin 2 (*tfr2*), acyl-CoA synthetase long-chain family member 4b (*acsl4b*), lysophosphatidylcholine acyltransferase 3 (*lpcat3*), lipoxygenase (*lox*), cyclooxygenase (*cox*), glutamate cysteine ligase (*gcl*), glutathione peroxidase 4a (*gpx4a*), heat shock protein beta associated protein1 (*hspbap1*), sterol regulatory element binding transcription factor 1 (*srebp1*), stearoyl-CoA desaturase (*scd*), carnitine palmitoyl-transferase (*cpt*), cytochrome p450 family 7 subfamily B member 1(*cyp7b1*), nuclear factor kappa b (*nfκb*), and interleukin 1β/10/15 (*il-1β/10/15*)]. All qRT-PCR primers were synthesized by Shanghai Biotech Co., Ltd. (Shanghai, China). The qRT-PCR program consisted of a pre-denaturation step at 95 °C for 30 s, followed by 40 cycles of 95 °C (denaturation) for 5 s and 60 °C for 30 s, and finally 95 °C for 10 s. Relative mRNA expression was calculated using the $2^{-\Delta \Delta \mathrm{Ct}}$ method, with *β-actin* as a normalization reference gene [29].

### 2.7. Statistical Analysis

The data were analyzed via one-way analysis of variance (ANOVA) using the SAS 9.4 software (Institute Inc., Cary, NC, USA) following assessments for normality and homogeneity of variance using the Shapiro–Wilk and Levene’s tests. Duncan’s multiple comparison test was conducted among the groups, with a significant difference indicated by *p* < 0.05. All data were presented as mean ± standard error (SE), and visualizations were generated using GraphPad Prism 8.0 (GraphPad, La Jolla, CA, USA).

## 3. Results

### 3.1. Plasma Biochemical Parameters

**Figure 1:**
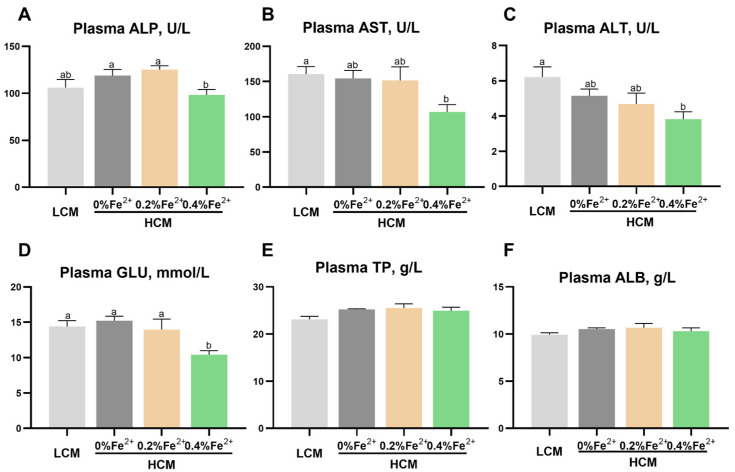
Effect of Fe^2+^ supplementation in the HCM diet on the plasma biochemical parameters of grass carp. (**A**) ALP, alkaline phosphatase; (**B**) AST, aspartate aminotransferase; (**C**) ALT, alanine aminotransferase; (**D**) GLU, glucose; (**E**) TP, total protein; (**F**) ALB, albumin. All data are expressed as mean ± standard error (SE). Different small letters above the bars indicate significant differences among groups (Duncan’s test, *p* < 0.05).

Following the eight-week feeding trial, the plasma ALP concentration was significantly lower in the HCM + 0.4%Fe^2+^ group compared to the HCM and HCM + 0.2%Fe^2+^ groups (*p* < 0.05, Figure 1A). However, this difference was not statistically significant between LCM and HCM + 0.4%Fe^2+^ groups (*p* > 0.05). Compared to the LCM group, AST and ALT levels were significantly lower in the HCM + 0.4%Fe^2+^ group (*p* < 0.05, Figure 1B,C). However, there were no significant differences between the HCM and HCM + 0.2%Fe^2+^ groups (*p* > 0.05). The plasma GLU content in the HCM + 0.4%Fe^2+^ group showed a notable decrease compared to the other groups (*p* < 0.05, Figure 1D). However, there were no significant differences in plasma TP and ALB levels among the experimental groups (*p* > 0.05, Figure 1E,F).

### 3.2. Hepatic Lipid-Metabolism-Related Indices

**Figure 2:**
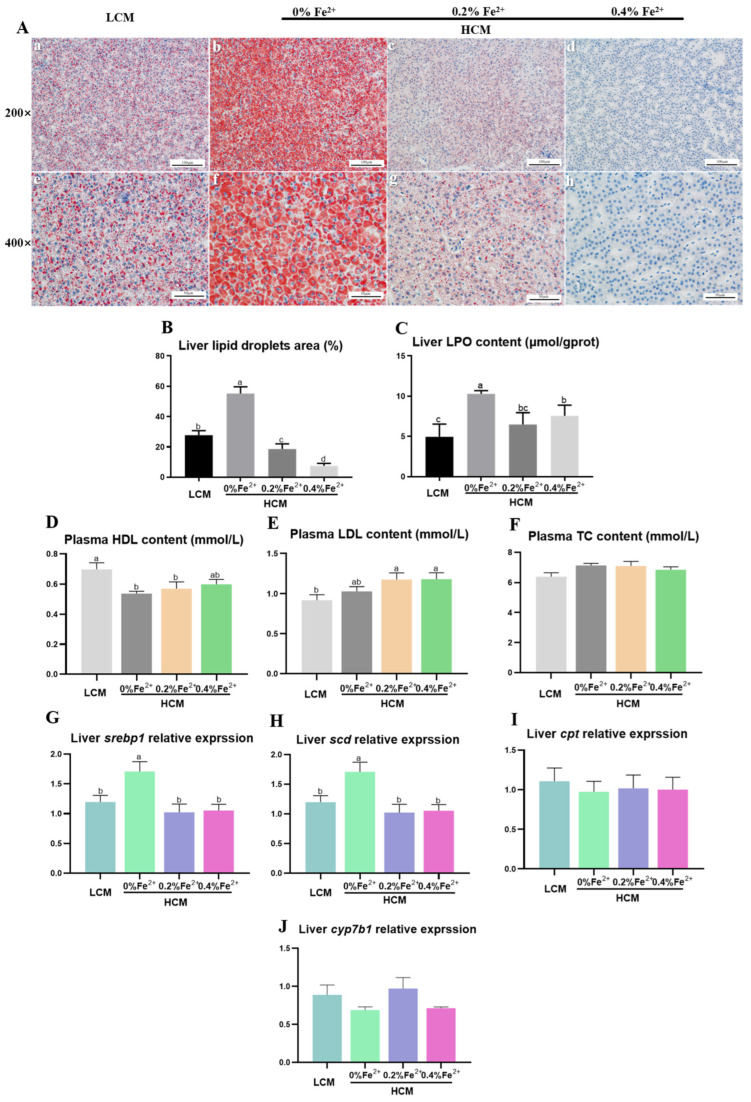
Effects of Fe^2+^ supplementation in HCM diet on hepatic and plasma lipid metabolism of grass carp. (**A**) Morphology of hepatic adipocytes using Oil Red O staining. a–d: Microscopic magnification 200× hepatic adipocyte morphology; e–h: Microscopic magnification 400× hepatic adipocyte morphology. The lipid droplets are stained in red, whereas the nuclei are stained in blue. (**B**) Quantification of the relative area of lipid droplets; (**C**) LPO, lipid peroxide; (**D**) HDL, high-density lipoprotein; (**E**) LDL, low-density lipoprotein; (**F**) TC, total cholesterol; (**G**) *srebp1*, sterol regulatory element binding transcription factor 1; (**H**) *scd*, stearoyl-CoA desaturase; (**I**) *cpt*, carnitine palmitoyl-transferase; (**J**) *cyp7b1*, cytochrome p450 family 7 subfamily B member 1. All data are expressed as mean ± standard error (SE). Different small letters above the bars indicate significant differences among groups (Duncan’s test, *p* < 0.05).

The results of Oil Red O staining in liver tissues revealed a significantly larger lipid droplet area in the HCM group compared to both the LCM and Fe^2+^-supplemented HCM groups (*p* < 0.05, Figure 2A,B). Moreover, with increasing Fe^2+^ supplementation, the lipid droplet area significantly decreased, with the HCM + 0.4%Fe^2+^ group exhibiting a notably lower lipid droplet area compared to the HCM + 0.2%Fe^2+^ group (*p* < 0.05). The hepatic LPO content was significantly decreased in the LCM group and Fe^2+^-supplemented HCM groups compared with that of the HCM group (*p* < 0.05, Figure 2C). In contrast, there was no significant difference between the HCM + 0.4%Fe^2+^ and HCM + 0.2%Fe^2+^ groups (*p* > 0.05). Plasma HDL level was significantly lower in the HCM and HCM + 0.2%Fe^2+^ groups compared to the LCM group (*p* < 0.05, Figure 2D). It was not significantly different, although a slight increase was observed in the HCM + 0.4%Fe^2+^ group compared to the HCM and HCM + 0.2%Fe^2+^ groups (*p* > 0.05). Moreover, the plasma LDL level of the HCM group was slightly higher than those of the LCM group, albeit not significantly (*p* > 0.05, Figure 2E), whereas the Fe^2+^-supplemented HCM groups exhibited a significant increase (*p* < 0.05). The mRNA expressions of *srebp1* and *scd* in the liver of the HCM group were significantly upregulated compared with that of the LCM group and Fe^2+^-supplemented HCM groups (*p* < 0.05, Figure 2G,H). There were no significant differences in *cpt* and *cyp7b1* expression levels in the liver among the experimental groups (*p* > 0.05, Figure 2I,J).

### 3.3. Expression of Iron-Transport-Related Genes in the Liver

**Figure 3:**
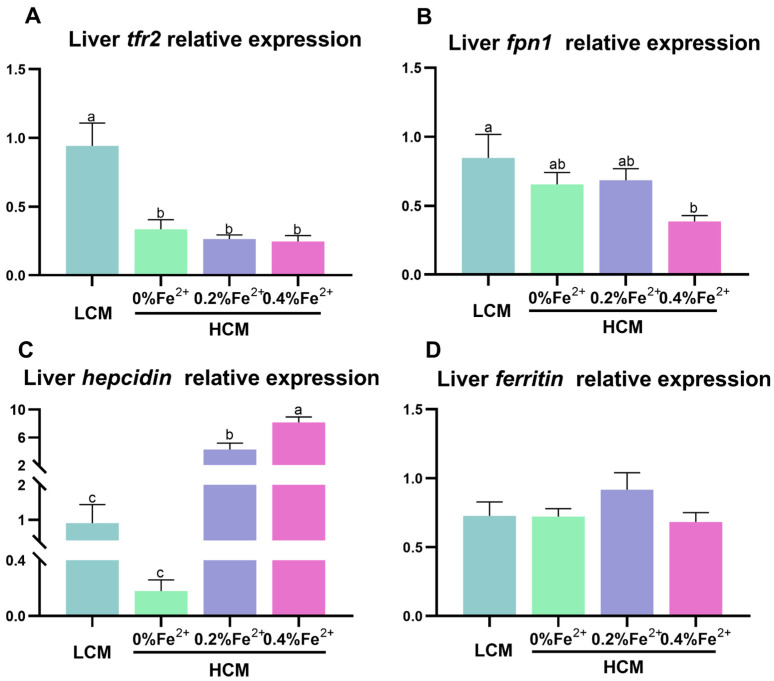
Effect of Fe^2+^ supplementation in HCM diet on gene expressions related to iron transportation in grass carp liver. (**A**) *tfr2*, transferrin 2; (**B**) *fpn1*, ferroportin-1; (**C**) *hepcidin*; (**D**) *ferritin*. All data are expressed as mean ± standard error (SE). Different small letters above the bars indicate significant differences among groups (Duncan’s test, *p* < 0.05).

*Trf2* expression was significantly down-regulated in the HCM group and Fe^2+^-supplemented HCM groups compared to the LCM group (*p* < 0.05, Figure 3A). Compared with the LCM group, there was a significant reduction in *fpn1* expression in the 0.4% Fe^2+^-supplemented HCM group (*p* < 0.05, Figure 3B). *Hepcidin* expression was significantly up-regulated in response to Fe^2+^ supplementation in a dose-dependent manner. Specifically, 0.4% Fe^2+^ supplementation induced significantly higher *hepcidin* expression than 0.2% Fe^2+^ supplementation (*p* < 0.05, Figure 3C), whereas 0.2% Fe^2+^ supplementation resulted in significantly higher *hepcidin* expression compared to the HCM group (*p* < 0.05). No significant differences in *ferritin* expression were observed among the experimental groups (*p* > 0.05, Figure 3D).

### 3.4. Expression of Ferroptosis-Related Genes in the Liver

**Figure 4:**
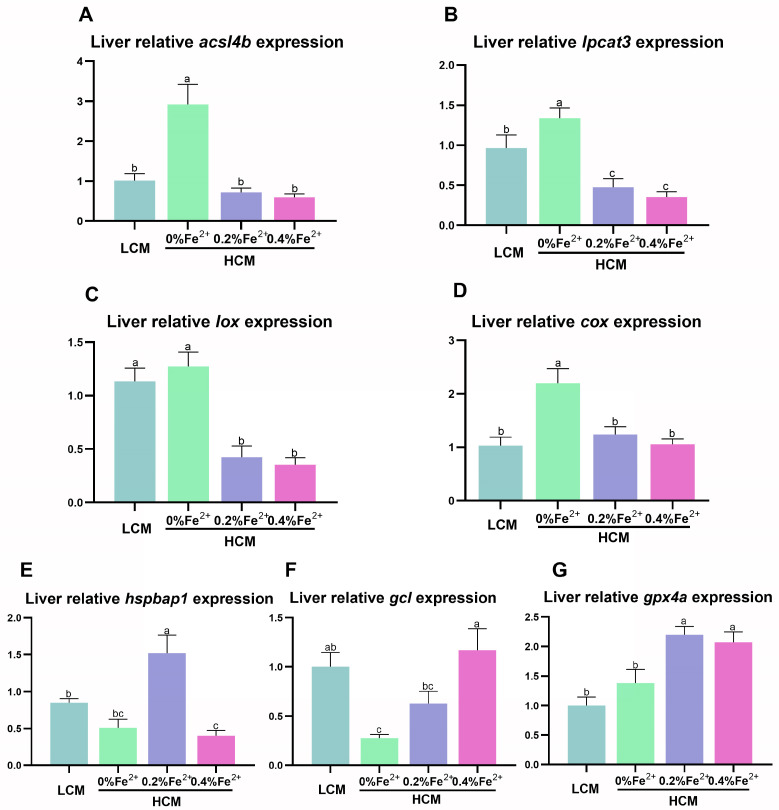
Effect of Fe^2+^ supplementation in HCM diet on the expression of genes related to ferroptosis in grass carp liver. (**A**) *acsl4b*, acyl-CoA synthetase long chain family member 4b; (**B**) *lpcat3*, lysophosphatidylcholine acyltransferase 3; (**C**) *lox*, lipoxygenase; (**D**) *cox*, cyclooxygenase; (**E**) *hspbap1*, heat shock protein beta associated protein1; (**F**) *gcl*, glutamate cysteine ligase; (**G**) *gpx4a*, glutathione peroxidase 4a. All data are expressed as mean ± standard error (SE). Different small letters above the bars indicate significant differences among groups (Duncan’s test, *p* < 0.05).

The HCM group exhibited significantly higher *acsl4b*, *lpcat3*, and *cox* expression compared to the other groups (*p* < 0.05, Figure 4A,B,D). Notably, *lpcat3* and *lox* expression was significantly lower in the Fe^2+^-supplemented HCM groups compared to the LCM group (*p* < 0.05, Figure 4B,C). Moreover, the expression of *hspbap1* was significantly higher in the HCM group supplemented with 0.2% Fe^2+^ compared to the remaining experimental groups (*p* < 0.05, Figure 4E). The expression of *gcl* in the 0.4% Fe^2+^ supplemented HCM group was significantly higher than that of the HCM group with or without 0.2% Fe^2+^ supplementation (*p* < 0.05) but was not significantly different from that in the LCM (*p* > 0.05, Figure 4F). Additionally, the expression of *gpx4a* in the HCM groups with Fe^2+^ supplementation was significantly higher than that in the LCM and HCM groups (*p* < 0.05, Figure 4G).

### 3.5. Expression of Inflammation-Related Genes in the Liver

**Figure 5:**
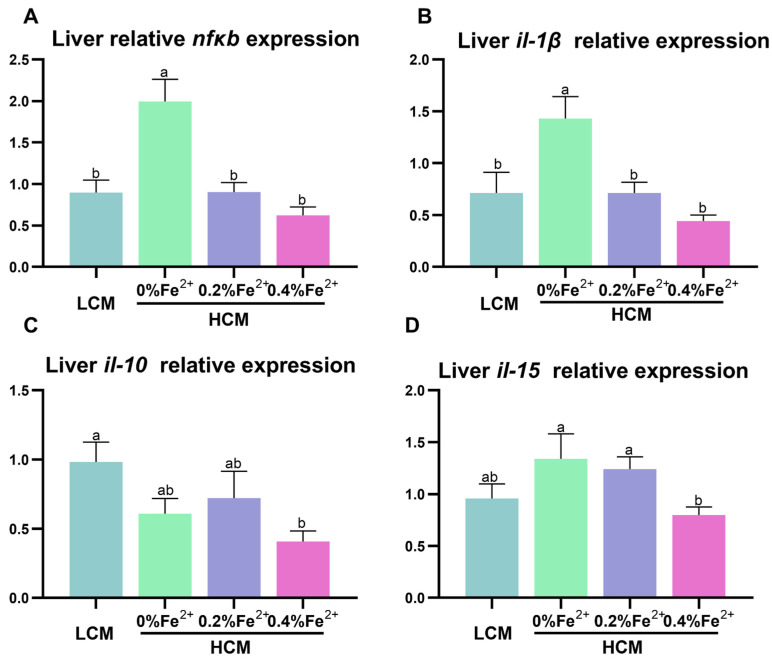
Effect of Fe^2+^ supplementation in HCM diet on the expression of genes related to inflammation in grass carp liver. (**A**) *nfκb*, nuclear factor kappa b; (**B**–**D**) *il-1β/10/15*, interleukin-1β/10/15. All data are expressed as mean ± standard error (SE). Different small letters above the bars indicate significant differences among groups (Duncan’s test, *p* < 0.05).

The expressions of *nfκb* and *il-1β* were significantly up-regulated in the HCM group compared to all the other groups (*p* < 0.05, Figure 5A,B). *Il-10* expression was significantly inhibited in the HCM + 0.4%Fe^2+^ group compared to the LCM group (*p* < 0.05, Figure 5C) and did not differ from the HCM and HCM + 0.2%Fe^2+^ group (*p* > 0.05). Additionally, *il-15* expression in the HCM + 0.4%Fe^2+^ group was significantly lower than that in the HCM and HCM + 0.2%Fe^2+^ groups (*p* < 0.05, Figure 5D), but was not different from that in the LCM group (*p* > 0.05).

## 4. Discussion

Excessive amounts of CSM in the diet can lead to oxidative stress, primarily due to the high content of FG in the HCM diets [7,33]. The addition of FeSO_4_ to feeds effectively reduces FG content, as Fe^2+^ binds to FG, forming a potent complex that detoxifies it [16]. Previous studies on grass carp and laying hens have demonstrated that FG can cause liver damage and exacerbate fat deposition in hepatocytes [7,34]. In our study, the HCM diet impacted plasma lipid levels, resulting in decreased HDL content and increased LDL content. This effect was subsequently reversed by the supplementation of Fe^2+^ in the HCM diet. Elevated plasma LDL levels suggest an increased assembly of very low-density lipoprotein particles in the liver, promoting the transport of TG to the bloodstream [35]. Conversely, reduced HDL levels indicate impaired hepatic reverse cholesterol transport. Interestingly, there was no significant change in the plasma HDL concentrations in the 0.4% Fe^2+^ supplementation group. This result suggests that the effect of Fe^2+^ supplementation on plasma lipid metabolism in HCM may be dose-dependent. The lipid deposition in fish liver gradually intensified with increased CSM levels in feed [36,37]. Consistent with these reports, our study demonstrated that the HCM diet exacerbated hepatic fat deposition, which was alleviated by Fe^2+^ supplementation in the HCM diet. Elevated plasma LDL concentrations were observed in the Fe^2+^-supplemented HCM group, supporting the reduced lipid accumulation in the liver. Analysis of gene expression patterns revealed that the genes related to lipid synthesis (*srebp1* and *scd*) were significantly upregulated in the liver of grass carp fed the HCM diet. However, this upregulation was inhibited by Fe^2+^ supplementation (0.2% and 0.4% FeSO_4_·7H_2_O). The transcription factor *srebp1* plays a dominant role in regulating lipid homeostasis, and *scd1* is a downstream signaling factor of *srebp1* in the AMPK signaling pathway [38,39,40,41]. *Srebp1* acts as an activator of *scd1* and promotes fatty acid synthesis, thereby regulating adipogenesis [42]. Disruptions in liver lipid metabolism often lead to hepatic injury [36]. ALT is rapidly released into the bloodstream, and AST levels increase in response to liver cell damage [43]. Moreover, elevated GLU levels are often associated with oxidative stress [44]. In our previous study, supplementation with 0.4% FeSO_4_·7H_2_O in the HCM diet notably improved the growth performance of juvenile grass carps [7], and in the present study, plasma levels of ALP, AST, ALT, and GLU were reduced due to Fe^2+^ supplementation in the HCM diet. These findings indicate that FG in the feed contributes to disorders in hepatic lipid metabolism by inhibiting lipolytic genes and activating fat synthesis in the liver while also causing damage to the antioxidant system. However, these adverse effects can be ameliorated by Fe^2+^ supplementation in the HCM diet [34].

Disorders in iron metabolism can lead to ferroptosis, a condition characterized by the entry of Fe^3+^ into cells through binding to transferrin. Overactivation of transferrin-related proteins can result in intracellular iron overload [23]. Heat shock protein beta 1 (HSPB1) plays an important role in reducing intracellular iron content by inhibiting transferrin expression [45]. In this study, Fe^2+^ supplementation in the HCM diet inhibited intracellular iron uptake by upregulating *hspbap1* expression and downregulating *tfr2* expression. It is worth noting that fish fed the HCM diet also showed reduced *tfr2* expression. This might be attributed to the preferential binding of Fe^2+^ to FG, resulting in less binding to *tfr2*. The exact regulatory mechanism of this phenomenon was further elucidated in the present study. Hepcidin binds to fpn1 to ubiquitinate it, which is subsequently endocytosed into the lysosome for degradation, thereby maintaining cellular iron transport homeostasis [46,47,48]. Similarly, we observed that Fe^2+^ supplementation in the HCM diet increased *hepcidin* expression and decreased *fpn1* expression. The mechanism of iron homeostasis regulation suggests that in response to elevated iron levels in vivo, *hepcidin* expression is upregulated, thereby attenuating intestinal iron absorption [49,50]. These results suggest that Fe^2+^ supplementation in the HCM diet helps maintain intracellular iron homeostasis by reducing iron uptake and efflux. Lipid peroxidation as a susceptibility factor for ferroptosis. Our previous and present results demonstrated that MDA and LPO levels in the liver of fish fed the HCM diet were significantly increased, indicating increased lipid peroxidation [7]. GPX4 is an important enzyme responsible for removing peroxides and hydroxyl radicals generated by LOX and PUFAs, thereby reducing lipid peroxidation [51,52]. In cases of acute ammonia exposure, hepatic *gpx4* expression was suppressed, leading to the accumulation of lipid peroxides and the induction of ferroptosis [53]. In contrast, ACSL4, LPCAT3, and LOX are involved in fatty acid metabolism and promote lipid peroxidation, which are key factors affecting ferroptosis [54]. Higher transcription levels of *acsl4* and *lpcat3* and lower transcription levels of *slc7a11* and *gpx4* in the liver of yellow catfish indicated induced ferroptosis due to excess iron [49]. In our study, Fe^2+^ supplementation in the HCM diet alleviated hepatic ferroptosis mediated by lipid peroxidation. This effect was attributed to Fe^2+^ modulating the expression levels of key genes associated with ferroptosis, including the downregulation of *acsl4b*, *lpcat3*, and *lox*, and the upregulation of *gpx4a* and *gcl* levels.

**Figure 6:**
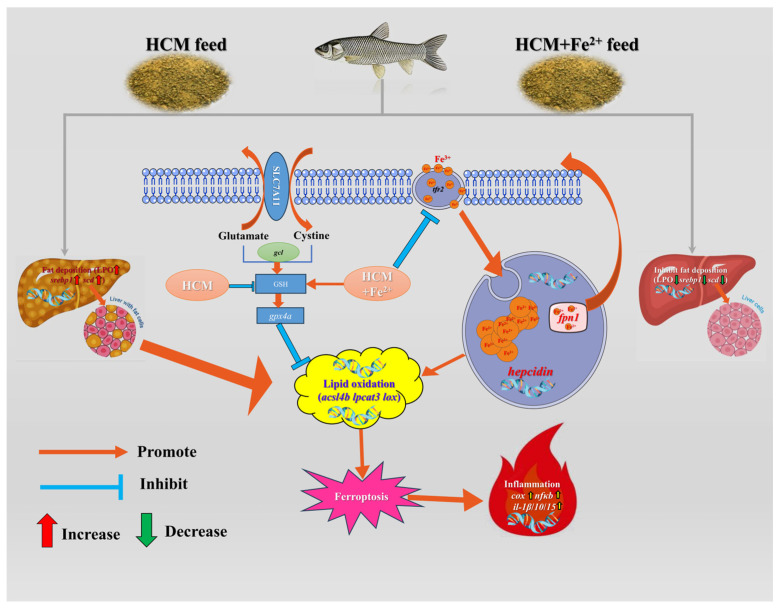
Regulation mechanisms of Fe^2+^ supplementation on HCM diet-induced lipid metabolism disorders and ferroptosis. Recombinant solute carrier family 7, member 11 (SLC7A11); lipid peroxide (LPO); sterol regulatory element binding protein (*srebp1*); stearoyl-CoA desaturase (*scd*); glutamate cysteine ligase (*gcl*); glutathione (GSH); glutathione peroxidase 4a(*gpx4a*); acyl-CoA synthetase long-chain family member 4b (*acsl4b*); lysophosphatidylcholine acyltransferase 3 (*lpcat3*); lipoxygenase (*lox*); transferrin 2 (*tfr2*); ferroportin-1 (*fpn1*); *hepcidin*; cyclooxygenase (*cox*); nuclear factor kappa b (*nfκb*); Interleukin-1β/10/15 (*il-1β*/*10*/*15*).

Ferroptosis is characterized by membrane rupture and is accompanied by the release of DAMPs, which trigger strong inflammatory reactions [55]. Inflammatory responses in fish triggered by FG in the feed have been reported extensively [7,56,57]. *Nfκb*, being the most important transcription factor in the inflammatory response, activates downstream target genes such as *cox2* and other inflammatory genes [58]. *Cox2* is an inducible enzyme encoded by prostaglandin endoperoxide synthase 2 (PTSG2) and can activate macrophages and other inflammatory cells [59,60]. Additionally, *cox2* suppresses *gpx4* activity through phospholipid oxidation and promotes ferroptosis [21]. Our study demonstrated that the HCM diet activated the *nfκb*/*cox* signaling pathway and increased inflammatory factor transcription levels (*il-1β/10/15*). However, Fe^2+^ supplementation alleviated the inflammatory response. Polyethylene exposure exacerbates liver injury by promoting ferroptosis and activating the *nfκb/cox2* pathway [61]. These results suggest that the HCM diet can trigger an inflammatory response by inducing ferroptosis, but it can be alleviated by Fe^2+^ supplementation (Figure 6).

## 5. Conclusions

Collectively, our findings demonstrated that the addition of Fe^2+^ to an HCM diet (38% CSM) helped maintain iron transport homeostasis in the liver of juvenile grass carps, inhibited the occurrence of ferroptosis, reduced liver lipid deposition, and consequently alleviated the inflammatory response induced by the high content of CSM dietary (Figure 6).

## Figures and Tables

**Table 1:** Ingredients and nutrient composition of experimental diets (air-dried basis).

Ingredients	Diets			
	LCM	HCM	HCM + 0.2%Fe^2+^	HCM + 0.4%Fe^2+^
Fish meal ^1^	53.3	53.3	53.3	53.3
Soybean meal ^1^	215.9	64.8	64.8	64.8
Rapeseed meal ^1^	162.2	0	0	0
Cottonseed meal ^1^	163.1	384.6	384.6	384.6
Cottonseed protein concentrate ^2^	42.2	84.5	84.5	84.5
	177.2	177.2	177.2	177.2
Rice bran	120.1	120.1	120.1	120.1
Wheat bran	0	49.5	47.5	45.5
Soybean oil	20	20	20	20
Calcium dihydrogen phosphate	10	10	10	10
Mineral premix ^3^	5	5	5	5
Vitamins premix ^3^	5	5	5	5
Vitamin C (55%)	5	5	5	5
Choline chloride	5	5	5	5
Microcrystalline cellulose	10	10	10	10
Bentonite	6	6	6	6
FeSO_4_·7H_2_O			2	4
Total	1000	1000	1000	1000
Nutrient contents				
Crude protein (%)	37.14	39.67	39.62	39.35
Crude lipid (%)	7.45	7.24	7.30	7.45
Ash (%)	8.53	8.66	8.75	8.79
Gross energy (MJ/kg)	19.69	19.60	19.64	19.61
Fe (mg/kg)	740	700	1100	1500
Free gossypol (mg/kg)	222	406	416	403

Notes: ^1^ Provided by Wuxi Tongwei Co., Ltd. (Wuxi, China), free gossypol content in cottonseed meal was approached to 1275 mg/kg. ^2^ Provided by Xinjiang Jinlan Plant Protein Co., Ltd. (Shihezi, China). ^3^ Mineral and vitamins premix provided by Wuxi Hanove Animal Health Products Co., Ltd. (Wuxi, China). Mineral premix (IU, g or mg/kg of diet): calcium biphosphate, 20 g; sodium chloride, 2.6 g; potassium chloride, 5 g; magnesium sulphate, 2 g; ferrous sulphate, 0.9 g; zinc sulphate, 0.06 g; cupric sulphate, 0.02 g; manganese sulphate, 0.03 g; sodium selenate, 0.02 g; cobalt chloride, 0.05 g; potassium iodide, 0.004 g. Vitamins premix (IU, g or mg/kg of diet): vitamin A, 25,000 IU; vitamin D3, 20,000 IU; vitamin E, 200 mg; vitamin K3, 20 mg; thiamin, 40 mg; riboflavin, 50 mg; calcium pantothenate, 100 mg; pyridoxine HCl, 40 mg; cyanocobalamin, 0.2 mg; biotin, 6 mg; folic acid, 20 mg; niacin, 200 mg; inositol, 1000 mg; vitamin C, 2000 mg; choline, 2000 mg.

**Table 2:** Primer sequences for qRT-PCR.

Genes	Primer (5′-3′)	Accession No./Reference	Product Length (bps)
*tfr2*	F: AGCTGGGATGGAGGAGACTT	XM_051881185.1	113
	R: AGGATGGCCTGATCCAGACT		
*hepcidin*	F: CAGCCGTTCCGTTCGTACA	Wei et al. (2018) [30]	192
	R: AGCCTTTGTTACGACAGCAG		
*ferritin*	F: TTGAGACACACTACTTGGACGAG	Yang et al. (2021) [31]	191
	R: GGCATGTAGGGCATTAAACACTC		
*fpn1*	F: GACCAGTTAACCAACATTCTGGC	Yang et al. (2021) [31]	197
	R: TCCTGGTCATCAGTTTCCTTCTG		
*acsl4b*	F: CGTCTGATCTCGCAGTGGTT	XM_051895363.1	180
	R: CTGTCAGCTCCAGCACATGA		
*lpcat3*	F: TTACGCCGTCTCTGTTGGAG	XM_051864303.1	160
	R: CGCTTCATTGCTGGAACCAC		
*lox*	F: TTTGCCGTCAGGTATCGGTG	XM_051908919.1	111
	R: TGCAGCTGATCCGTGTGATT		
*cox*	F: TGTGGATGTGTTCAACCGCT	XM_051865758.1	197
	R: GCTTCCTGTTCTTGCCTGGA		
*gpx4a*	F: TTATCCATCGCGTCTTGCTGT	XM_051907667.1	100
	R: TAAATGGATGTGGCCGTCTGC		
*gcl*	F: ACGAATCGGACCACTTCGAG	XM_051916883.1	177
	R: TCACACGGGTGAGAAGAACG		
*hspbap1*	F: CGAGTGCACACCGTTACTCT	XM_051904930.1	141
	R: TACTCTGGCCTCATCGTCCA		
*srebp1*	F: AGAAACTGCCCATCAATCGC	XM_051886061.1	189
	R: TCCTCAACACTGCCGACTTATT		
*scd*	F: GTTTGTGCCCTGGTTCTT	XM_051914897.1	154
	R: GGGGTTAATGGTGCTGTC		
*cpt*	F: GCCACTGTAAAGGAGAACC	XM_051899001.1	272
	R: GGATGCCTCATAAGTCAAG		
*cyp7b1*	F: ACGGTACTTATTGCAGGGAG	XM_051916835.1	135
	R: TGGGTAATCGAACGTCCTGG		
*nfκb*	F: AGTCCGATCCATCCGCACTA	XM_051918333.1	85
	R: ACTGGAGCCGGTCATTTCAG		
*il-1β*	F: AGAGTTTGGTGAAGAAGAGG	Xu et al. (2016) [32]	292
	R: TTATTGTGGTTACGCTGGA		
*il-10*	F: AACGAGAACGTGCAACAGAA	XM_051913375.1	101
	R: TGGACAGCTGTTGGCAGAAT		
*il-15*	F: TTGCCAATGGCTGAAGGTCA	XM_051909817.1	108
	R: TGGTGTGTACAAGCGTGCAT		
*β-actin*	F: CGTGACATCAAGGAGAAG	XM_051886219.1	215
	R: GAGTTGAAGGTGGTCTCAT		

## Data Availability

The original contributions presented in the study are included in the article and further queries can be directed to the corresponding author.
